# Professor Liebig

**Published:** 1861-07

**Authors:** 


PROFESSOR LIEBIG.—This eminent chemist has been appointed foreign associate of the Academy of Sciences of Paris, in consequence of the vacancy left by the death of Tiedemann.

